# Production of Nanocellulose by Enzymatic Treatment for Application in Polymer Composites

**DOI:** 10.3390/ma14092124

**Published:** 2021-04-22

**Authors:** Daria Zielińska, Kinga Szentner, Agnieszka Waśkiewicz, Sławomir Borysiak

**Affiliations:** 1 Institute of Chemical Technology and Engineering, Poznan University of Technology, Berdychowo 4, 60965 Poznan, Poland; daria.d.zielinska@doctorate.put.poznan.pl; 2 Department of Chemistry, Faculty of Forestry and Wood Technology, Poznan University of Life Sciences, Wojska Polskiego 75, 60625 Poznan, Poland; kinga.szentner@up.poznan.pl (K.S.); agnieszka.waskiewicz@up.poznan.pl (A.W.)

**Keywords:** nanocellulose, polypropylene composites, enzymatic modification, structure, nucleation activity, mechanical properties

## Abstract

In the last few years, the scientific community around the world has devoted a lot of attention to the search for the best methods of obtaining nanocellulose. In this work, nanocellulose was obtained in enzymatic reactions with strictly defined dispersion and structural parameters in order to use it as a filler for polymers. The controlled enzymatic hydrolysis of the polysaccharide was carried out in the presence of cellulolytic enzymes from microscopic fungi—*Trichoderma reesei* and *Aspergillus* sp. It has been shown that the efficiency of bioconversion of cellulose material depends on the type of enzymes used. The use of a complex of cellulases obtained from a fungus of the genus *Trichoderma* turned out to be an effective method of obtaining cellulose of nanometric dimensions with a very low polydispersity. The effect of cellulose enzymatic reactions was assessed using the technique of high-performance liquid chromatography coupled with a refractometric detector, X-ray diffraction, dynamic light scattering and Fourier transform infrared spectroscopy. In the second stage, polypropylene composites with nanometric cellulose were obtained by extrusion and injection. It was found by means of X-ray diffraction, hot stage optical microscopy and differential scanning calorimetry that nanocellulose had a significant effect on the supermolecular structure, nucleation activity and the course of phase transitions of the obtained polymer nanocomposites. Moreover, the obtained nanocomposites are characterized by very good strength properties. This paper describes for the first time that the obtained cellulose nanofillers with defined parameters can be used for the production of polymer composites with a strictly defined polymorphic structure, which in turn may influence future decision making about obtaining materials with controllable properties, e.g., high flexibility, enabling the thermoforming process of packaging.

## 1. Introduction

Cellulose is one of the most abundant naturally occurring polysaccharides and whose units are linked by β-1,4-glycosidic bonds [1,2]. This natural biopolymer is characterized by the presence of hydroxyl groups and strong hydrogen bonds, thanks to which cellulose has good physical and mechanical properties [3]. Moreover, cellulose is composed of amorphous and crystalline regions, the presence of which affects the modification and final properties of this material.

Using various methods, e.g., mechanical methods, hydrolysis using acids or ionic liquids and enzymatic hydrolysis, it is possible to obtain from cellulose a biodegradable material with nanometric dimensions—nanocellulose [3,4,5]. Nanocellulose has very interesting properties, ranging from low weight and density, high shape factor [6], high biocompatibility to hydrophilicity and very good mechanical properties, e.g., high tensile strength, high stiffness and high modulus of elasticity, which is greater than that of Kevlar fibers [3,7,8]. Due to these features, interest in this material is constantly growing. Due to the source of origin, extraction methods, morphology, the characteristic particle size, properties and crystallinity, there are several types of nanometric cellulose. Based on ISO standards, nanocellulose can be divided into two categories. The first is cellulose nanoobjects, in which cellulose nanocrystals (CNCs) and cellulose nanofibrils (CNFs) can be distinguished. The second type of nanocellulose is nanostructured cellulose, which includes cellulose microcrystals (CMCs), microcrystalline cellulose (MCC), cellulose microfibrils (CMFs), microfibrillated cellulose (MFC) and bacterial cellulose (BC) [3,8,9,10,11,12,13].

The oldest method of obtaining nanocrystalline cellulose is the acid hydrolysis of cellulose. During the hydrolysis process, the cellulose crystalline fractions remain intact, and the amorphous parts are decomposed [14]. The biggest disadvantage of this process is the reduction of the thermal stability of the CNC, which is related to the presence of sulfone groups on the surface of nanocrystals. Additionally, the separation of concentrated acid from nanostructures is very time-consuming and requires the use of special reactors [15,16,17,18].

Another method to obtain nanocrystalline cellulose is the ionic liquid (IL) technique. The solubility of cellulose in ionic liquids, as in the case of acid hydrolysis, is affected by the crystallinity of the cellulosic material. Due to the higher energetic stability and lower energy of the crystalline fractions, dissolving these areas is difficult [19,20]. Several disadvantages of using IL should also be mentioned, such as: difficulty in purifying compounds, high viscosity of ionic liquids or degradation of the cellulose chain [21].

In addition, to obtain nanometric cellulose, mechanical methods that rely on the application of high shear forces are also used [22,23]. Among the applied mechanical methods, there are pressure homogenization, the use of ultrasound or ball grinding and cryogenic grinding [3,9,14,22,24]. Mechanical methods find their application as techniques preceding the hydrolysis processes, thus supporting the efficiency of the reaction [25]. However, the main disadvantage of obtaining cellulose of nanometric size is the high energy consumption [3,23]. Moreover, the use of these operations may affect the morphology of cellulose fibers, consequently reducing its degree of crystallinity and obtaining a material with low repeatability of particles [24].

A relatively new method, thanks to which it is possible to obtain cellulose with a nanometric size, is the enzymatic hydrolysis of cellulose material. It is noteworthy that this method is characterized by high effectiveness [26,27], selectivity and a lower energy expenditure [28,29]. It is also worth noting that the enzymatic process eliminates the use of harmful reagents, which are used, e.g., in chemical methods, in favor of the use of biodegradable cellulases, which are neutral and, consequently, have no emissions of harmful chemicals that affect ecology and laboratory equipment [20,30]. Cellulose, due to the presence of a large amount of hydrogen bonds in it, is a material whose enzymatic modification requires a synergistic correlation of cellulolytic enzymes [31,32,33,34,35]. Scientists in their works have proved that the effectiveness of this complicated process is influenced not only by the conditions of the hydrolysis reaction, such as temperature, pH of the reaction environment [36] or the type, activity level and concentration of the selected substrate [37,38], but also the type or degree of polymerization of the cellulose used, its crystallinity and porosity [39,40]. Moreover, it was noticed that the efficiency of the modification process of this polysaccharide is influenced by the dispersion–morphological properties and polymorphism of the selected cellulose material [41,42,43,44].

Previous work on the enzymatic hydrolysis of cellulose has been conducted towards obtaining simple sugars [45,46] or nanocellulose for applications in medicine, pharmacy and cosmetology [47,48].

In recent years, there has been an increasing interest in renewable lignocellulosic fillers in the production of polymer composites. Composites with a polymer matrix reinforced with lignocellulose additives are known [49,50,51,52,53,54,55], as well as with a filler in the form of micrometric cellulose [14,56,57,58], although in recent years more and more attention has been focused on nanocellulose [9,59,60,61,62,63]. Polymer composites containing nanometric particle filler can have very good strength characteristics [9,64,65], strong barriers to gases and water vapor [66] and transparency [67], which determine possible applications in the packaging, construction, automotive and medical industries [6,60,65,68,69,70,71,72].

Particular interest has been focused on the context of the application potential of systems of polypropylene with nanocellulose [61,73,74,75,76,77]. Sojoudiasli et al. [77] proved in their work that the produced polypropylene systems with 2 wt. CNCs are characterized by much better mechanical properties compared to unfilled polymer, although the proper dispersion of nanofillers in the polymer matrix plays an extremely important role [75]. Similar conclusions came from Ljungberg et al. [73], who strengthened the polypropylene matrix with nanocellulose, and thanks to which they obtained a nanocomposite with satisfactory strength parameters, emphasizing that the quality of the filler dispersion plays a key role in the final properties of the tested systems. However, according to Liu et al. [61], the addition of nanocellulose to the polypropylene matrix significantly increased the aging resistance of the composite systems tested. Based on the review of the available literature, it can be concluded that the use of nanocellulose as a filler for polymer matrices results in obtaining very good strength parameters of nanocomposites, and, consequently, unique properties of the final materials. It is worth noting that in the work to date, nanocellulose used in the production of polypropylene nanocomposites has been obtained mainly by chemical methods, e.g., acid hydrolysis, or by the use of mechanical treatment. Alternatively, scientists working with nanocellulose/polypropylene systems used a commercial cellulose material with a nanometric particle size. Moreover, in these publications, no attempts were made to analyze the particle size and the polydispersity, while the work was mainly focused on the mechanical properties of nanocomposite systems.

According to our knowledge, there is a lack of research related to the production of polypropylene composites containing nanometric cellulose obtained as a result of enzymatic reactions, which enable the production of nanoparticles. Therefore, the aim of this study was to carry out a controlled enzymatic hydrolysis of cellulose with the use of enzymes of various activities in order to obtain a filler of nanometric size. This study also included a detailed analysis of the influence of the obtained cellulose nanofillers on the supermolecular structure, phase transitions, nucleation activity and mechanical properties of the obtained composite materials. It is worth adding that the influence of nanometric cellulose on the formation of the polymorphic structure of polypropylene matrix, which consequently allows one to obtain composite materials with strictly defined strength characteristics, has not been analyzed.

## 2. Materials and Methods

### 2.1. Materials

Micrometric cellulose Sigmacell Type 20 (Cel_A) with an average particle size of 20 μm and micrometric cellulose Sigmacell Type 101 (Cel_B) with an average particle size of 18 ± 3 μm were purchased from Sigma-Aldrich (Poznań, Poland).

Cellulases from the microscopic fungus *Trichoderma reesei* ATCC 26,921 with activity ≥700 U/g and an enzyme complex from the fungus *Aspergillus* sp. used under the trade name Viscozyme^®^ L with activity ≥100 U/g were purchased from Sigma-Aldrich (Poznań, Poland). The used cellulolytic enzymes also differed in the composition of carbohydrates. The *Trichoderma reesei* enzyme complex includes two exoglucanases that degrade the cellulose chain from two extreme ends, at least five endoglucans attacking the biopolymer in the middle of the chain and cellobiase. The following carbohydrates occur in the cellulolytic enzyme obtained from the microscopic fungus *Aspergillus* sp.: arabanase, cellulase, β-glucanase, hemicellulase and xylanase.

All reagents used for the enzymatic hydrolysis of cellulose, e.g., citric acid, trisodium citrate, were purchased from Sigma-Aldrich (Poznań, Poland).

Isotactic polypropylene (PP) with the trade name Moplen HP500 N (MFI_230 °C/2.16 kg–_ 2.4–3.2 g/10 min, isotactic–95%, T_m_ = 163–164 °C) manufactured by Basell Orlen Polyolefins (Płock, Poland) was used as a polymer matrix.

### 2.2. The Process of Enzymatic Treatment of Cellulose

**Figure 1:**
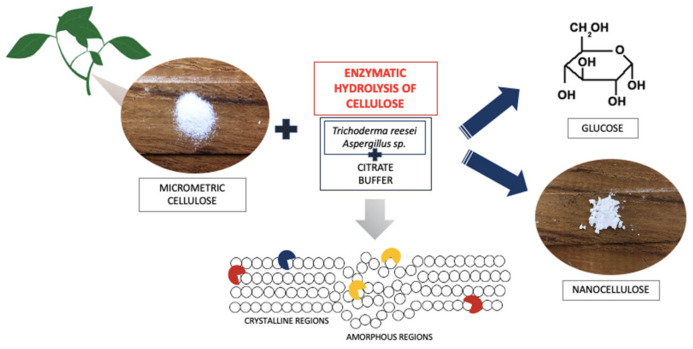
Scheme of the process of enzymatic hydrolysis of cellulose.

To 200 mg of both types of microcrystalline celluloses used (Cel_A and Cel_B) were added 4 mL of a citrate buffer solution with a concentration of 50 mM and pH = 4.8, which was prepared by mixing appropriate amounts of citric acid and trisodium citrate. Pre-incubation of the samples for 30 min was performed at 50 °C with a shaking speed of 150 rpm/min (Incubated Shaker, Lab Companion, JeioTech, Korea). Next, 2 mL of the mixture containing cellulolytic enzymes diluted 1:50 by volume in citrate buffer were added. The actual 24 h incubation was performed at 50 °C with a shaking speed of 250 rpm/min. The modified samples were stored at −20 °C until chromatographic analysis was performed. The process of enzymatic treatment of cellulose is shown in Figure 1.

Table 1 shows the designations of the obtained cellulose materials.

### 2.3. Characteristics of Cellulose Fillers after the Enzymatic Treatment Process

#### 2.3.1. Glucose Analysis by Liquid Chromatography

High-performance liquid chromatography coupled with a refractometric detector (HPLC/RI) was used for the quantitative analysis of glucose—the main product of the enzymatic hydrolysis of cellulose. Samples after enzymatic hydrolysis were centrifuged at 1000 rpm/min for 15 min, and the supernatant was filtered through a 0.20 μm chromatographic filter (Chromafil Pet 20/15 MS, Macherey-Nagel, Steinheim, Germany) before chromatographic analysis. Glucose concentration was analyzed using a 2695 Waters high-performance liquid chromatograph (HPLC) system with a 2414 Refractive Index (RI) Detector (Waters, Milford, MA, USA). A Bio-Rad Aminex HPX-87H column (Bio-Rad, Woodinville, WA, USA) was used with the HPLC system operating at a column temperature of 65 °C. The mobile phase was 0.5 mM H_2_SO_4_ with a flow rate of 0.6 mL/min. The quantification of glucose was performed by measuring the peak areas at the retention time according to the relevant calibration curve. Data were processed using Empower^TM^ 1 software (Waters, Milford, MA, USA).

#### 2.3.2. X-ray Diffraction (XRD)

The XRD technique was used to determine the effect of enzymatic modification on the crystal structure of cellulose filler. Cellulose material was analyzed using a TUR M-62 X-ray diffractometer (Carl Zeiss AG, Jena, Germany) using a copper anode with a wavelength of Cu Kα 1.5418 Å as an X-ray source in the angle range of 2 θ = 5–30° with a counting step of 0.04°/3 s with excitation of 30 kV and 25 mA. The deconvolution of the peaks was performed using the method proposed by Hindeleh and Johnson [78], improved and programmed by Rabiej [79]. The degree of crystallinity (X_c_) of the cellulose fillers was calculated, i.e., the quotient of the content of the crystalline fraction (A_cr_) to the sum of the amorphous fraction (A_am_) and the crystalline fraction, using Formula (1):

(1) $$\mathrm{Xc}=\frac{{\Sigma \;A}_{\mathrm{cr}}}{{\Sigma \;A}_{\mathrm{cr}}+{\;A}_{\mathrm{am}}}\times 100\%$$

#### 2.3.3. Dynamic Light Scattering

The dynamic light scattering (DLS) technique was used to measure the particle size and dispersion properties of cellulosic materials based on the non-invasive back scattering (NIBS). A Zetasizer Nano ZS-90 (Malvern Instruments Ltd., Malvern, UK) operating in the range of 0.6 to 6000 nm was used for DLS analysis. Measurement parameters were as follows: a laser wavelength of 663 nm, a medium viscosity of 2.004 cP, medium refractive index of 1.337, a measurement temperature of 25 °C. Before each measurement, cellulose material weighing 0.01 g was dispersed in 25 cm^3^ of propanol and then homogenized using ultrasound for 20 min, and then it was placed in a cuvette. The particles within the fluid exhibited Brownian motion, which made the measurement possible.

#### 2.3.4. Fourier Transform Infrared Spectroscopy (FTIR) Analysis of Cellulose

The structural changes in cellulose after enzymatic treatment was confirmed by the FTIR spectroscopy. The spectra were obtained using a pelleting technique—1 mg cellulose was mixed with 200 mg bromide potassium (KBr, Sigma-Aldrich, Steinchein, Germany). Spectra were registered using an FTIR Nicolet iS5 spectrometer (Thermo Fisher Scientific, Madison, WI, USA) at a range from 4000 to 500 cm^−1^ at a resolution of 4 cm^−1^, registering 16 scans.

### 2.4. Obtaining Polymer Nanocomposites

The production process of nanocomposite systems involved of two stages. The first consisted in mixing cellulose filler with isotactic polypropylene in a single-screw extruder (Fairex, Le Bourget, France), with an extrusion speed 150 rpm/min and with a plasticizing temperature from 160 to 200 °C, which was dependent on the extrusion zones. Then, the composite materials containing 1 wt. percentage of the cellulose fillers were injected using an injection molding machine (ENGEL 80/25 HLS, ENGEL Austria GmbH, Schwertberg, Austria) with mold temperature 30 °C and an injection speed of 90 mm/s. The obtained samples were stored in a desiccator for 48 h.

### 2.5. Characteristics of Composite Materials

#### 2.5.1. Structural Investigations (XRD)

Diffractometric tests were performed, taking into account the same measurement conditions as in the case of the celluloses described above. The research was carried out to determine the supermolecular structure of composite materials, with particular emphasis on the effect of cellulose fillers on polymorphic changes in the polymer matrix. After separating the diffraction curves, the amorphous part and the baseline, the content of polymorphs of the polymer matrix was determined using the Turner–Jones formula [80], where I_α1_ + I_α2_ + I_α3_ are the intensities of the peaks derived from the α form, while I_β1_ is the peak intensity from the β form. k in Equation (2) means the content of the polymorphic form β in polypropylene. The distribution of individual peaks from both polymorphs is shown in Section 3.2.1.

(2) $$k=\frac{I_{\beta 1}}{I_{\beta 1}+\left(I_{\alpha 1}+I_{\alpha 2}+I_{\alpha 3}\right)}$$

#### 2.5.2. Differential Scanning Calorimetry

The method of differential scanning calorimetry (DSC) was used to investigate the influence of enzymatic modification of cellulose on the course of phase transformations occurring in composite systems.

The polymer composites were analyzed using a Netzsch DSC 200 scanning calorimeter (Netzsch Group, Selb, Germany). In the first part of the tests, the samples were subjected to a heating process at a rate of 10 °C·min^−1^ in an argon atmosphere from 40 °C to 200 °C, which was kept at this temperature for 3 min. In the next stage, the test material was cooled at a rate of 5 °C·min^−1^ to a temperature of 40 °C. The process was carried out in two experimental cycles in order to reduce the thermal memory of the analyzed samples, and the data for the second pass of composite materials were used to analyze the results. For the tested systems, the following kinetic parameters were determined: melting point (T_m_), enthalpy of the melting process (ΔH_m_) and crystallization temperature (T_c_) as the highest temperature of exothermic peaks. Moreover, by analyzing the data from the second pass, the half-time of the crystallization process (t_0.5_) was determined, which is defined as the time during which 50% conversion of amorphous areas to the crystalline phase occurs. For this purpose, the following relationship was used (3), in which the degree of phase conversion (α) was calculated by integrating the enthalpy (H) of the exothermic transformation over time (t) [81]:

(3) $$\alpha =\frac{\int_{0}^{t}\left(\frac{\mathrm{dH}}{\mathrm{dt}}\right)\mathrm{dt}}{\int_{0}^{\alpha }\left(\frac{\mathrm{dH}}{\mathrm{dt}}\right)\mathrm{dt}}$$

#### 2.5.3. Hot-Stage Polarized Light Microscopy

Hot-stage polarized light microscopy was used to observe isothermal crystallization of PP in the presence of cellulose fillers.

For this purpose, a Labophot-2 polarizing optical microscope (Nikon, Tokyo, Japan) coupled with a Panasonic CCS camera (Panasonic, Kadoma, Japan) and the Linkam TP93 thermal attachment (Linkam, Tadworth, UK) was used. The analyzed material (in film form) was subjected to two research cycles. The first one involved heating the sample to a temperature of 200 °C at a rate of 40 °C·min^−1^. The second one consisted in cooling the composite films at a rate of 20 °C·min^−1^, where the analysis of the isothermal crystallization process was performed at a temperature 136 °C. By analyzing microscopic examinations, the kinetics of the crystallization process (induction time, the rate of formation of transcrystalline structures) and the phenomena occurring at the interface of composite systems were determined.

#### 2.5.4. Tensile Tests

Tensile tests were carried out in at least eight repetitions for each test. For this purpose, a testing machine with a 20 kN load cell (Zwick Z020, Zwick/Roell, Ulm, Germany) was used with the following parameters: temperature 23 °C, relative humidity approx. 50%, stretching speed 5 mm/min. The conducted mechanical tests allowed for the analysis of such strength properties of nanocomposite materials as: Young’s modulus, elongation at break, tensile strength and impact strength.

## 3. Results and Discussion

### 3.1. Characterization of Cellulosic Fillers after Enzymatic Treatment

#### 3.1.1. HPLC Analysis of Glucose

Research using high-performance liquid chromatography coupled with a refractometric detector was used to determine the bioconversion efficiency of cellulose materials during enzymatic hydrolysis reactions. Table 2 shows the results of the qualitative and quantitative analysis of glucose as the major product of the enzymatic hydrolysis reaction by HPLC/RI. The obtained results constitute the mean value of three independent replications.

On the basis of the obtained results, it can be seen that the monosaccharide content obtained as a result of cellulose bioconversion depends on the type of cellulolytic enzymes used. The highest efficiency was observed for the hydrolysis reactions catalyzed with a cellulolytic enzyme with higher activity (≥700 U/g) obtained from the microorganism of the genus *Trichodema* for cellulose A (Cel_A-Tr) and cellulose B (Cel_B-Tr). On the other hand, the use of an enzyme complex derived from the fungus *Aspergillus* sp. (≥100 U/g) in the case of both tested materials (cellulose A and B) resulted in a much lower efficiency of the hydrolysis reaction, as the glucose concentration was at a low level, in the range of 3.66–4.30 mg/mL.

Moreover, when comparing both cellulose materials treated with the same enzyme complex (Cel_A-Tr and Cel_B-Tr), it can be noticed that cellulose A is more susceptible to bioconversion than cellulose B. In cellulose bioconversion, the efficiency of the process is significantly influenced by both the composition of the cellulase complex, and the structural conditions of the material subjected to hydrolysis. The obtained outcomes are the result of the fact that in the initial stage of hydrolysis, amorphous areas are degraded and, in the next stage, crystalline ones, which was previously described in the literature [82,83]. The changes shown in the chromatographic analysis can be explained by the fact that for cellulose B, which is largely amorphous, the bioconversion is more dynamic in the initial stages of the process. However, in further stages, the efficiency of the process may decrease due to the small proportion of crystalline regions in Cel_B cellulose. In addition, the observed effect of this enzyme complex may also indicate the accumulation of reaction-inhibiting products, which in turn translates into lower efficiency of Cel_B hydrolysis. If the enzyme complex with *Trichoderma* is used, high activity in the crystalline regions is also possible, which in turn leads to greater efficiency of the cellulose bioconversion process and to a higher glucose content for Cel_A-Tr compared to Cel_B-Tr. Moreover, there is a noticeable lack of clear differences in the effectiveness of the applied enzyme complex with *Aspergillus* sp., which follow firstly from the lower activity of the complex itself, and also from a different, less effective composition of the complex. Cellulases from *Aspergillus* sp. have more β-glucosidases than other enzyme classes that are important in the hydrolysis process, e.g., endo-exoglucanases, compared to the enzyme complex of *Trichoderma reesei* [84], which in turn translates into a low efficiency of the process with the use of this enzyme. However, in the case of using enzymes derived from *Aspergillus* sp., no clear differences were observed in the yield of the reaction between the materials used.

It is also worth emphasizing that obtaining a large amount of nanometric cellulose fractions in the case of using the enzyme of *Trichoderma reesei* with high activity is associated with low recovery of nanocellulose, at the level of approx. 20%, and approx. 80% of microcrystalline cellulose conversion went to glucose (Table 2). In the case of using a cellulolytic enzyme obtained from the microscopic fungus *Aspergillus* sp. with lower activity, the bioconversion of cellulose to glucose is limited and, consequently, a higher efficiency of the cellulose filler is obtained. On the basis of the obtained results for this system, high recovery of nanocellulose was obtained, at a level of approx. 90% (Table 2), although the filler particles were characterized by a smaller amount of nanometric fractions and higher polydispersity, which will be discussed in the Section 3.1.3. The obtained results indicate that the selection of enzyme complexes and the supermolecular structure of cellulose are of decisive importance for the design of cellulose enzymatic processes.

#### 3.1.2. XRD Investigation of Cellulose after Enzymatic Treatment

**Figure 2:**
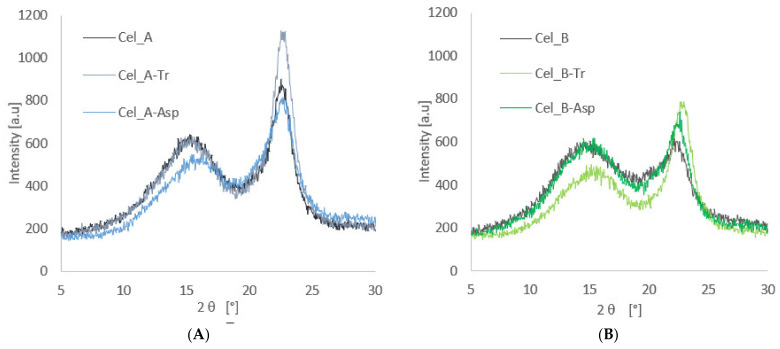
Diffractograms of cellulose Cel_A (**A**) and Cel_B (**B**) and celluloses hydrolyzed with enzymes.

XRD studies were used to analyze the supermolecular structure of native celluloses and enzymatic hydrolyzed celluloses. Figure 2 shows the diffraction patterns for cellulosic materials before and after the biotransformation process.

The X-ray analysis for both tested materials showed the presence of the polymorphic form cellulose I at 2 θ angles of 15°, 16.5° and 22.7° [85], whose peaks correspond to lattice planes with Miller indices amounting to (0$\bar{1}$0), (110) and (200) [86]. However, the treatment of cellulosic material with enzymes resulted in a marked differentiation of the intensity of the diffraction peaks, which indicates changes in the content of the crystalline phase.

The results obtained for Cel_A cellulose show that enzymatic hydrolysis with the use of cellulases from the microscopic fungus *Trichoderma reesei* (Cel_A-Tr) is responsible for the greatest changes in the supermolecular structure, which confirms that the highest intensity of the diffraction curve was obtained (approx. 1100). In the case of microcrystalline native cellulose, the intensity is approx. 850. The performed calculations showed an increase in the degree of crystallinity for the Cel_A-Tr system to the value of 56%. In turn, the use of the *Aspergillus* sp. enzyme reduced the degree of crystallinity to 43% (Table 3). Similar relationships were observed in the case of Cel_B cellulose, for which the enzymatic treatment with the *Trichoderma reesei* enzyme resulted in a significant increase in the degree of crystallinity (from 29% to 51%).

The enzymatic hydrolysis of cellulose has a complicated mechanism in which all types of enzymes take part, i.e., exogluconases, β-glucosidases and endoglucanases [87]. The first group of enzymes is responsible for the gradual degradation of cellulose into cellobiosis, starting at the ends of the chains. β-glucosidases hydrolyze soluble celodextrins to glucose during this time. A third group of enzymes randomly hydrolyze internal glycosidic linkages, shortening the cellulose chains. The observation of the increased crystallinity as a result of the action of *Trichoderma reesei* enzyme may be the result of the active activity of endoglucanases, which are responsible in the first stage for the degradation of cellulose chains in the amorphous fraction. This is in line with the work of Lindman et al. [19], who proved that amorphous regions from crystalline fractions are distinguished by a lower energy balance, which ultimately leads to easier dissolution of these parts. Additionally, in other studies, the depolymerization of cellulose leading to the shortening of polymer chains was observed [88,89,90]. An interesting result is the observation of a decrease in the degree of Cel_A crystallinity in the case of the use of the *Aspergillus* sp. enzyme, which can clearly indicate a low activity of this type of enzyme in relation to the degradation of cellulose chains. This may result from the type of enzymes used when this cellulolytic enzyme is used. It is worth emphasizing that the enzyme from *Aspergillus* sp. differs from the cellulase from the microscopic fungus *Trichoderma reesei* not only in activity, but also in constituent enzymes, whose synergistic correlation is responsible for the efficiency of the cellulosic material bioconversion process and, consequently, for changes in structure of the tested material. Moreover, Houfani et al. [91], Pirich et al. [92], Arantes et al. [33] and Albornoz-Palma et al. [32] also emphasized in their work that the effectiveness of enzymatic cellulose hydrolysis is influenced by properly selected cellulases, which should synergistically correlate with each other.

However, in the case of Cel_B cellulose hydrolysis in the presence of the *Aspergillus* sp. enzyme, a slight increase in crystallinity can be observed, which may indicate a greater interference of this enzyme during the interaction with cellulose with a higher amorphous fraction content.

The obtained results correlate perfectly with the chromatographic results, in which it was also noticed that the use of *Trichoderma reesei* enzymes is the most effective in the context of the cellulose hydrolysis process, which is confirmed by the largest amount of glucose obtained.

#### 3.1.3. Determination of Particle Sizes for Treated Celluloses

**Figure 3:**
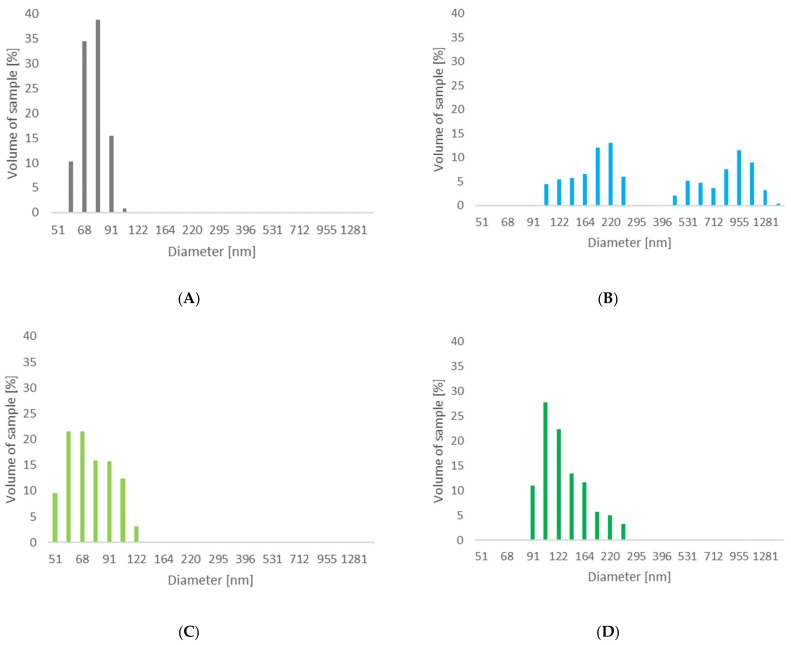
The particle size distribution of celluloses after enzymatic modification: (**A**) Cel_A-Tr, (**B**) Cel_A-Asp, (**C**) Cel_B-Tr, (**D**) Cel_B-Asp.

The laser diffraction technique was used to determine the effect of the enzymatic treatment of two types of native celluloses on the particle size. Figure 3 shows graphs illustrating the obtained results of particle sizes achieved for cellulose materials subjected to enzymatic hydrolysis with the use of the enzymes of *Trichoderma reesei* and *Aspergillus* sp.

The unmodified Cel_A cellulose material had a particle size of 20 μm. By using the enzymatic treatment of this cellulose with the use of cellulases from the microscopic fungus of the genus *Trichoderma*, clear changes in the nanometric structure content of the material were noticed, obtaining about 99% of the nanometric fraction below 100 nm in size, while the Z-average is 75 nm (Table 4). In the case of the analyzed cellulose modified with the *Aspergillus* sp. enzyme, there was a clear differentiation in the size of the material particles. For this material, no nanometric particles below the size of 100 nm were found, while particles were recorded in two fractions, in the range of 106–255 nm (53.3%) and 0.45–1.48 μm (46.7%). Additionally, this material has a Z-average value of 505 nm.

The enzymatic treatment of Cel_B cellulose with *Trichoderma reesei* also resulted in obtaining cellulose with a nanometric fraction below 100 nm at the level of approx. 84% and a Z-average equal 76 nm. For the cellulose material, after the enzymatic hydrolysis process with the enzyme from *Aspergillus* sp., a wide size range of the fraction was determined (91–255 nm). For this system, only a small number of nanometric particles (approx. 11%), and a Z-average equal 135 nm can be observed, although no micrometric fraction was detected. An interesting result in the case of the enzymatic process using cellulase from *Aspergillus* sp. is obtaining a much larger amount of Cel_B nanometric fractions compared to Cel_A cellulose. These results confirm that, e.g., the structure of cellulose material has a key influence on the course and efficiency of cellulose bioconversion reactions. Based on the literature [82,83], it is known that the crystalline regions in cellulose with an ordered structure and with lower energy are responsible for the stability of the chains and their high resistance to enzymatic decomposition, while the amorphous regions characterized by a disordered structure are more susceptible to enzymatic hydrolysis. Consequently, cellulose B (Cel_B), with a much larger number of amorphous regions, is more susceptible to the action of the enzyme agent *Aspergillus* sp., which therefore led to a smaller particle size compared to cellulose A, containing a greater number of regions with a crystalline structure.

It is also worth emphasizing that the enzymatic hydrolysis of all types of cellulose resulted in obtaining nanocelluloses with polydispersity (PDI) in the range of 0.105–0.217. According to the literature data [93], these values signal that the considered systems are moderately polydisperse. It should be noted that nanocelluloses obtained with the use of the *Trichoderma reesei* enzyme are characterized by lower polydispersion compared to fillers obtained with cellulase from *Aspergillus* sp.

Observing the obtained results of the particle size in our work, it should be emphasized that in other works, much larger particle sizes were obtained. Ribeiro et al. [94], using the *Carezyme* enzyme, obtained cellulose particles larger than 400 nm. Additionally, in the work of Beltramino et al. [95], nanocellulose, obtained by acid hydrolysis and enzymatically pretreated with cellulose provided by Fungal Bioproducts, had a Z-average of about 80–90 nm. In the work of Yarbrough et al. [46], as a result of the use of two types of enzymes (*T. reesei* and *Caldicellulosiruptor bescii*), cellulose particles were obtained in the Kraft pulp enzymatic hydrolysis process in two fractions with a size of approx. 100–200 nm and 500–700 nm. These works did not analyze the polydispersity of the obtained nanocellulose particles.

The conducted DLS studies perfectly correlate with our earlier chromatographic analysis, during which a significantly higher concentration of monosaccharide in the post-reaction material was noted for samples modified with cellulase from the microscopic fungus *Trichoderma reesei*, thanks to which it is possible to find a much higher efficiency of the process than in the case of the *Aspergillus* sp. Structural studies showed an increase in the degree of crystallinity of celluloses in the case of enzymatic hydrolysis with the enzyme of *Trichoderma reesei*, which confirms the effect of this type of enzyme towards the degradation of cellulose chains in the amorphous fraction. Moreover, the observations made prove that the efficiency of the enzymatic hydrolysis process is influenced by the type of cellulolytic enzyme used and the selection of a material with appropriate initial structural parameters.

#### 3.1.4. FTIR Spectroscopy

Infrared spectroscopy studies were used to evaluate the structural changes in cellulosic materials treated with cellulolytic enzymes.

**Figure 4:**
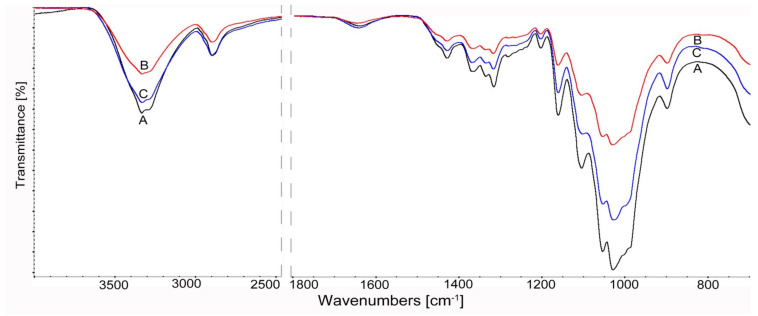
FTIR spectra of cellulose Cel_A after enzymatic reaction in the range 4000–500 cm^−1^: **A** Cel_A, **B** Cel_A-Tr, **C** Cel_A-Asp.

The highest susceptibility to bioconversion for the materials used was observed for cellulose A (Cel_A-Tr) after enzymatic treatment with the use of a complex of cellulases obtained from a fungus of the genus *Trichoderma*. For the vibrations of the hydroxyl group at about 3350 cm^−1^, a decrease in intensity and a narrowing of the bandwidth were found as well as a shift towards lower values of the wavenumber (Figure 4). These changes may result from intermolecular interactions and energy changes within the hydrogen bond during cellulose bioconversion [96].

The effect of enzymatic treatment for Cel_A cellulose is also illustrated by the “fingerprint” range of 1800–500 cm^−1^. Namely, there is a visible decrease in the intensity of CH deformation bands at 1370 cm^−1^, CH2 bands at C-6 at 1316 cm^−1^ and vibrations of δ COH in the plane at C-6 at 1199 cm^−1^. The reduction of the relative absorbance of the bands also applies to the deformation vibrations δ for the CH2 groups at C-6, at C6 δ OCH at 1429 cm^−1^. Its position is also attributed to changes in the rotation energy of hydroxyls at positions 3 and 6 (C3-O3 and C6-O6) [97]. This band also provides information about changes under the influence of enzymes in the crystalline regions of cellulose. The significant reduction in the intensity of this band is especially evident after the action of the cellulase complex of *Trichoderma reesei* Cel_A-Tr compared to the samples treated with *Aspergillus* sp. Cel_A-Asp (Figure 4).

Significant activity of cellulolytic enzymes was also found for the bands concerning amorphous areas of cellulose at 898 cm^−1^. An increase in the relative intensity of the 898 cm^−1^ band and a decrease at 1428 cm^−1^ for the Cel_A-Asp sample after treatment with *Aspergillus* sp. cellulases indicate a decrease in the crystallinity of this material. On the other hand, the different relationships observed of these bands for Cel_A-Tr cellulose with *Trichoderma reesei* show an increase in cellulose crystallinity after bioconversion. This is explained by the fact that the amorphous regions that are more susceptible to enzymes are destroyed first and then the crystalline regions are degraded. The results of the spectral analysis for cellulose A correlate with the results of the X-ray analysis (Table 3) and confirm the more effective action of cellulases obtained from *Trichoderma reesei*.

The influence of enzymes on structural changes is also reflected in vibrations of other bands, including those originating from the glycosidic bond. For Cel_A cellulose, a decrease in the intensity of the asymmetric stretching vibrations (antisymmetric bridge stretching) of the C–O–C glycosidic bond, as well as the stretching of C–O and deformation of OH in C–OH for the band at 1165 cm^−1^, was found. This indicates that the cellulose chain may be depolymerized under the influence of enzymes. It is also evidenced by the reduction of the relative absorbance of the C–O stretching vibrations of the ring in the plane at about 1098 cm^−1^.

The obtained results indicate that the use of cellulases from the fungus *Trichoderma reesei* effectively affects both amorphous and crystalline regions and the glycosidic bonding of cellulose, increasing the bioconversion effect of this material.

**Figure 5:**
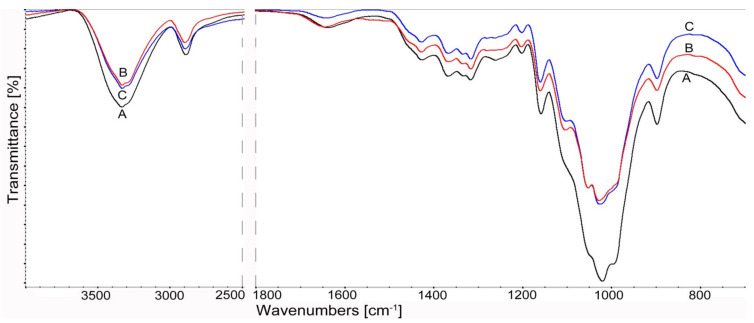
FTIR spectra of cellulose Cel_B after enzymatic reaction in the range 4000–500 cm^−1^: **A** Cel_B, **B** Cel_B-Tr, **C** Cel_B-Asp.

Structural changes and depolymerization of the cellulose chain after the action of enzyme complexes were also found for Cel_B cellulose. The spectral analysis presented in Figure 5 shows a decrease in the relative intensity of the OH hydroxyl stretching groups at about 3350 cm^−1^ and the CH vibration of the methyl and methylene groups at about 2897 cm^−1^. On the other hand, in the field of the “fingerprint”, the differences concern mainly the reduction of the intensity of the glycosidic bond at 1160 cm^−1^, and also the vibrations of the COC, CCO, CCH stretching bands at C-5 and C-6 at 898 cm^−1^. For Cel_B cellulose, these changes are also visible in the action of the enzyme complex from *Aspergillus* sp., mainly due to the interaction of cellulases with a material characterized by a large proportion of amorphous areas. It should be emphasized that, unexpectedly, no differences were found in the intensity of the peaks for Cel_B-Tr and Cel_B-Asp. This may have been caused by a completely different supermolecular structure of this type of cellulose, which due to the large number of amorphous areas will be characterized by a significantly different accessibility to the enzymes used. This is confirmed by DLS research. In the case of celluloses B (Cel_B-Tr and Cel_B-Asp), nanometric particles were obtained in a similar range of approx. 255 nm, without the participation of the micrometric fraction, and this can explain the obtaining of similar peak intensities on FTIR spectra. In the case of systems with cellulose A, a completely different behavior can be observed. Cel_A-Tr was characterized only by the nanometric size, and in the case of the Cel_A-Asp system, two fractions of cellulose particles—nanometric and micrometric—were found. The performed FTIR spectral analysis confirms and complements the X-ray and chromatographic analysis as well as DLS tests.

### 3.2. Characteristics of Composite Materials

#### 3.2.1. Structural Studies of Polymer Composites

**Figure 6:**
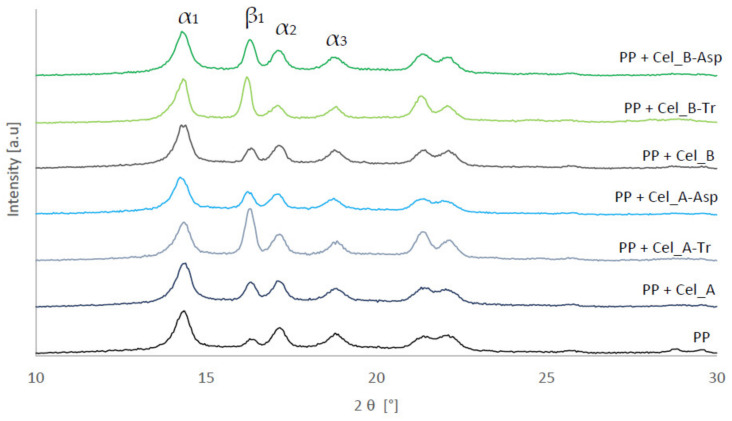
Diffractograms of polypropylene matrix and composite materials.

Structural studies of polymer composites were performed in order to determine the effect of controlled enzymatic modification of cellulose filler on their crystal structure. Figure 6 shows the XRD pattern of the polypropylene matrix and composites with cellulose filler before and after enzymatic treatment.

The presented diffractograms show the presence of two polymorphs—α and β—which are characteristic of the polypropylene matrix. This is evidenced by the occurrence of diffraction maxima for the α-PP form at the 2 θ angle equal to 14, 17, 18.5, 21 and 22° and for β-PP equal to 16.2°. Moreover, the illustrated changes in the intensity of the maximum corresponding to the β form of the polypropylene matrix on the XRD pattern indicate structural changes in the analyzed composite materials, which may consequently affect their strength properties. Table 5 shows the content of the β form for each of the tested systems.

The conducted diffractometric studies allowed us to determine the amount of the β form for each of the analyzed materials using the Turner–Jones formula [80]. For unfilled polymer, the content of β-PP was 10%, the value of which is characteristic for injection processes of semicrystalline materials in which shear forces occur. The systems with unmodified microcrystalline filler had similar values of this form, at the level of approx. 17–20%. The marked intensification of the β-PP variety for composites whose filler has been subjected to controlled enzymatic hydrolysis using cellulase from the microscopic fungus *Trichoderma reesei*, where the volume of this polymorphic variety fluctuates around 40%, is noteworthy. Moreover, the composite material with the filler after the treatment with the *Aspergillus* sp. enzyme was also characterized by an increase in the content of β-PP, amounting to approx. 22–29%, although its amount was definitely lower than in the case of other tested samples.

Structural investigations of composites perfectly correlate with the previously discussed results of laser diffraction. Polypropylene composites containing fillers subjected to enzymatic modification with the fungus of the genus *Trichoderma*, which were characterized by a higher content of the nanometric fraction, are characterized by a clearly greater content of the β polymorph in comparison to other tested systems. It can be concluded that the greater the number of nanometric particles in the analyzed material, the more likely it is to obtain higher shear forces during processing. Consequently, these composite systems have a larger amount of β-PP. A large number of studies can be found in the literature to present the effect of the particle size of the filler on the shear stress value or rheological properties [98,99,100]. In these works, the authors proved that the smaller the size of the filler particles in the form of wood flour in the polymer matrix, the greater the shear stresses and the apparent viscosity of the tested materials that can be obtained. Similar conclusions were reached by Bose and Mahanwar [101], who observed an improvement in the rheological properties of polymer systems with the addition of a filler, which was characterized by a smaller particle size than other tested materials.

#### 3.2.2. Investigation of the Nucleation Activity of Composite Materials by Differential Scanning Calorimetry

**Figure 7:**
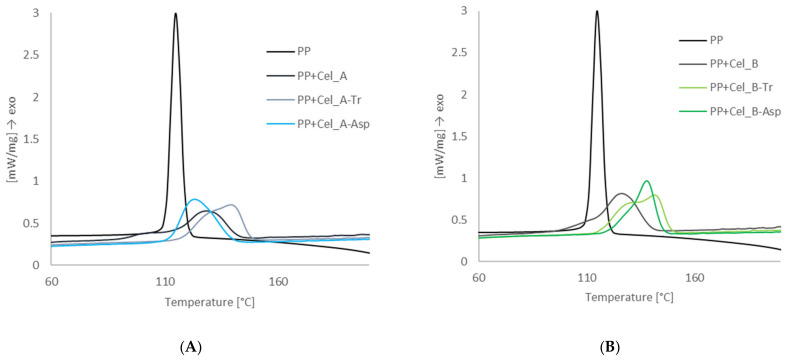
Thermograms of the crystallization process for polypropylene matrix and composite materials with cellulose Cel_A (**A**) and cellulose Cel_B (**B**) before and after modification.

Differential scanning calorimetry studies were carried out in order to determine the phase transformations taking place in composite materials containing microcrystalline cellulose and nanometric cellulose. Figure 7 shows the calorimetric curves of exothermic changes for a polypropylene matrix and composites containing Cel_A and Cel_B cellulose.

The presented calorimetric curves show the course of the crystallization process of the polypropylene matrix in the presence of cellulose fillers. For composite materials with an unmodified cellulose filler, the crystallization temperature values were 122.5 °C for the PP + Cel_A system and 122.1 °C for PP + Cel_B. It has been found that carrying out the enzymatic treatment of the cellulosic material is responsible for significant differences in the values of crystallization temperatures. The highest crystallization temperature was found for the systems modified with the enzyme of the genus *Trichoderma*. In both cases of the celluloses used, a significant increase in the T_c_ value by approx. 6–7 °C was observed, which oscillated at the level of 128.2 °C for PP + Cel_A-Tr and 129.2 °C for PP + Cel_B-Tr. A significant increase in the crystallinity temperature in the case of systems with modified cellulose with the *Trichoderma* enzyme can be explained by the nucleating effect of nanoparticles of this type of filler. According to Zanjanijam et al. [102], the increase in the crystallization temperature in composite systems may be caused by a reduction in the size of the filler particles and obtaining a homogeneous dispersion in the polypropylene matrix, which ultimately leads to an improvement in interfacial adhesion between the components. Moreover, Shabani et al. [103] proved that nanoparticles can affect the crystallinity of a polymeric material by changing the speed of growth and nucleation processes, which is explained by the effect of heterogeneous nucleation. For composites with a filler modified with cellulase from *Aspergillus* sp., an increase in the temperature value by approx. 5 °C was noted for PP + Cel_B-Asp, while for the material PP + Cel_A-Asp, a slight decrease in this T_c_ value by approx. 2 °C was observed when compared to composites containing native cellulose.

The tests of differential scanning calorimetry showed comparable values of the melting point for all composite systems, which are from approx. 162–166 °C.

**Figure 8:**
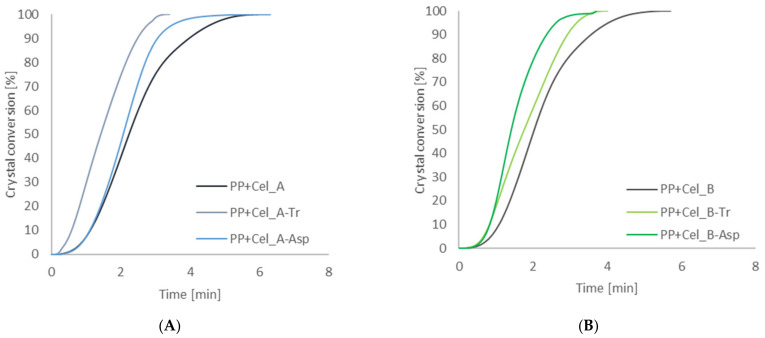
The crystal conversion of composites with PP matrix and unmodified and modified cellulose Cel_A (**A**) and unmodified and modified cellulose Cel_B (**B**).

The next stage of calorimetric tests was the determination of phase conversion curves for composites (Figure 8), on the basis of which the half-times for the crystallization process of composite systems were determined (Table 6).

The analysis of the obtained results showed that the action of specific cellulases on cellulose material, and consequently obtaining a filler with appropriate dispersion and morphological properties, have a significant impact on changes in the course of phase conversion curves of composite systems. The enzymatic treatment of the filler resulted in higher values of the degree of phase conversion and lower values of the half-times of the polypropylene matrix crystallization process in the presence of enzymatically modified fillers than in the case of composites with native cellulose. The most significant effect of enzymatic hydrolysis on cellulose material was observed with the use of cellulases from the microorganism *Trichoderma* in both systems, in which a clear reduction of crystallization half-times to the level of approx. 1.4 min was found. In the case of composite systems with a filler obtained after modification with cellulolytic enzyme from *Aspergillus* sp., a slight decrease in the value of crystallization half-times was noted compared to composite systems with native cellulose. However, in the case of celluloses modified with this type of enzyme, a shorter half-time of crystallization for Cel_B-Asp cellulose can be noticed. Moreover, the performed calorimetric tests allowed us to determine the value of the degree of crystallization of the polypropylene matrix and composites with native celluloses and with an enzymatically modified filler. The pure polymer matrix was characterized by a crystallinity degree value equal to 39%. The addition of cellulose fillers not subjected to enzymatic modification reactions and after the bioconversion process caused a significant decrease in the value of the degree of crystallization, obtaining comparable values for each of the systems, ranging from 20 to 25%.

On the basis of the obtained results, it can be concluded that the enzymatic treatment of the cellulose material is responsible for increasing the nucleic activity of the filler surface in the polypropylene matrix. However, it was shown that only in the case of enzymatic hydrolysis with the use of cellulolytic enzymes from the microorganism of the genus *Trichoderma* was a significant increase in nucleating activity obtained, which is manifested by an increase in the value of the degree of crystallinity, degree of phase conversion and reduction of half-times of crystallization. This can be explained by the fact that the action of the *Trichoderma* enzyme resulted in the production of a cellulose filler with a nanometric particle size, which is characterized by a much larger specific surface, which in turn causes a greater interaction at the interface with the polymer matrix. In the works [104,105], it can also be noticed that nanofillers are characterized by higher nucleating activity compared to micrometric fillers.

#### 3.2.3. Examination of the Morphology of Composite Materials by Microscopy with a Heating Attachment

Microscopic tests with a heating attachment were carried out in order to observe the phenomena at the interface in composite systems containing various types of cellulose fillers. The main goal was to analyze the influence of the enzymatic hydrolysis reactions on the process of shaping the crystal structure of the polymer matrix.

**Figure 9:**
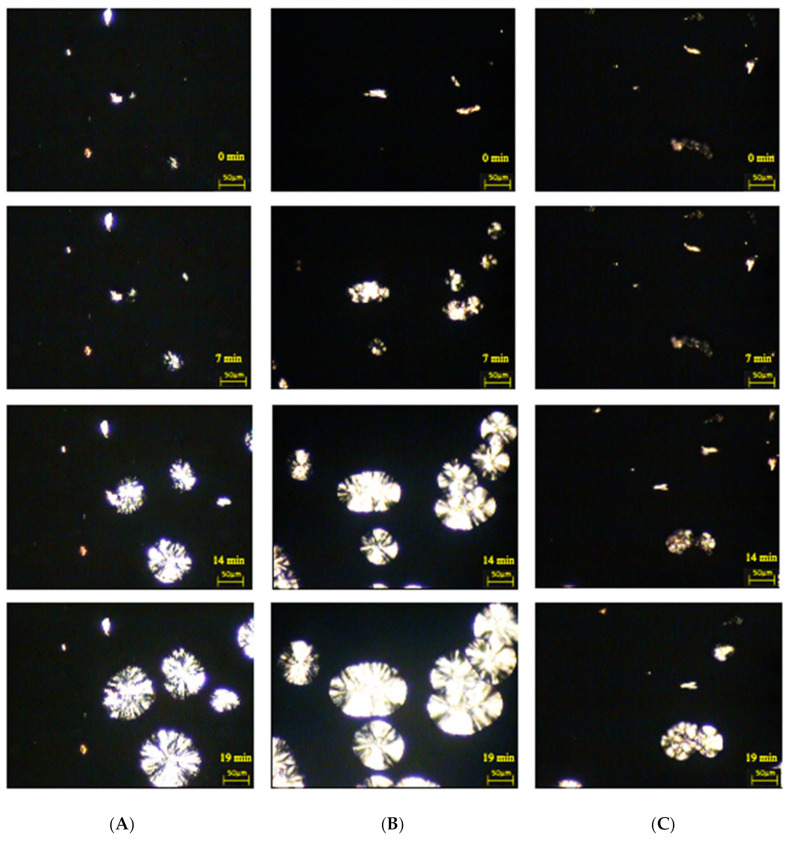
Microscopic images of the stages of nucleation processes and the increase in the crystal structure of isotactic polypropylene matrix (136 °C) in the presence of cellulose fillers: (**A**) PP + Cel_A, (**B**) PP + Cel_A-Tr, (**C**) PP + Cel_A-Asp.

**Figure 10:**
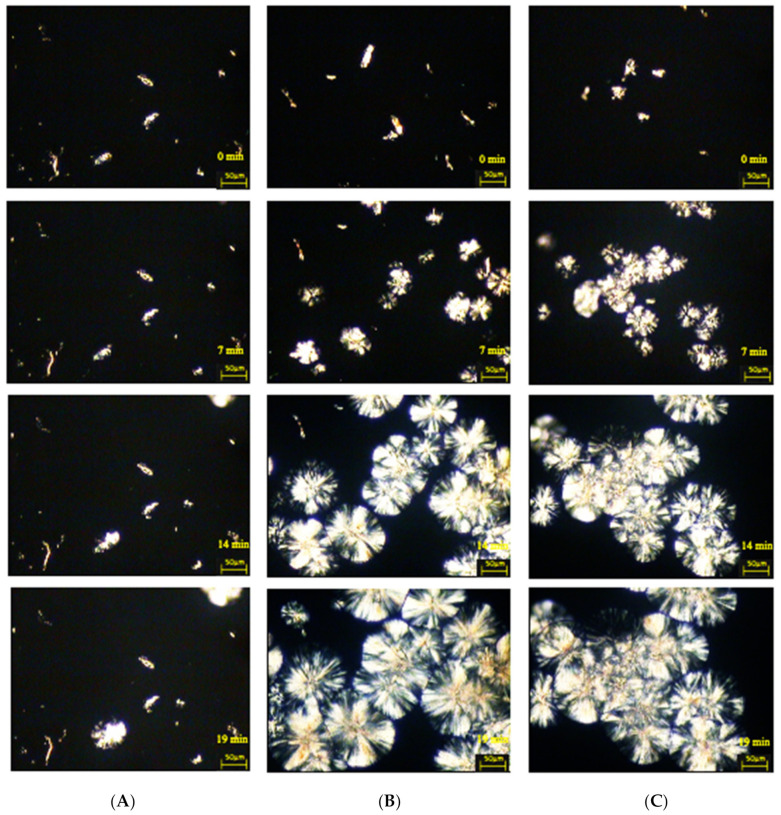
Microscopic images of the stages of nucleation processes and the increase in the crystal structure of isotactic polypropylene matrix (136 °C) in the presence of cellulose fillers: (**A**) PP + Cel_B, (**B**) PP + Cel_B-Tr, (**C**) PP + Cel_B-Asp.

Figure 9 and Figure 10 illustrate the isothermal crystallization process of cellulose composites.

All microscopic photos show composite systems that are characterized by the ability to form transcrystalline structures at the polymer–cellulose filler interface, albeit with different effectiveness of their formation. The nucleation ability of certain fillers is extremely high so that subsequent crystal growth is normal to the filler until the growing front is impeded by the growth of spherulites nucleated in the bulk. Thus, a columnar crystalline layer is known as a transcrystalline structure. It can be concluded that the effectiveness of the process of the formation of transcrystalline structures is influenced by the type of cellulose filler used.

In order to determine the nucleation capacity, kinetic parameters were determined: the time of induction of crystallization and the growth rate of TCL structures, which are presented in Table 6. In the case of composite materials containing nanocellulose after treatment with cellulase from the microorganism of the genus *Trichoderma* characterized by the shortest induction times (3 min), significantly higher values were found in the growth rate of the spherulitic structure both for the PP + Cel_A-Tr sample (2.8 μm/min) and for the PP + Cel_B-Tr material (3.2 μm/min). Moreover, when cellulose treatment with the cellulolytic enzyme from *Aspergillus* sp. was used, a significant deterioration in the ability to form transcrystalline structures on the filler surface for PP + Cel_A-Asp (1.2 μm/min) and an increase in induction time (11 min) were noted. For the Cel_B-Asp material, comparable values of the induction time (3 min) for composites with nanofillers after modification with the cellulolytic enzyme of *Trichoderma reesei* and an increase in the rate of formation of TCL structures (2.4 μm/min) were found. Obtaining a much lower crystallization growth rate and increasing the induction time in the case of the PP + Cel_A-Asp system may be correlated with the size of the filler particles. In the case of the Cel_A-Asp filler (as the only one), the presence of a significant number of micrometric particles (almost 50%) was found, which may significantly affect the nucleating effect in the polypropylene matrix. We also noted in our earlier work [106] that the increase in the micrometric particle content in the hybrid lignin filler is responsible for the deterioration of the nucleation capacity in the polypropylene matrix. This can be explained by the smaller specific surface, which in turn reduces the nucleation activity.

The formation of transcrystalline structures proves that there are large interactions between the polymer matrix and the filler. Based on the literature, it can be seen that carrying out chemical modifications is often responsible for reducing the ability to create TCL structures [81,107,108,109]. However, carrying out the enzymatic hydrolysis reaction resulted in a significant increase in nucleating activity, albeit mainly when using the enzymes of *Trichoderma reesei*. Moreover, the obtained results of microscopic examinations perfectly correlate with calorimetric studies, which also confirmed the highest nucleation abilities for this type of system. Obtaining high nucleation activity of the obtained cellulose fillers as a result of enzymatic treatment is extremely important from the point of view of processing processes, because it shortens the technological cycle times. In addition, the presence of transcrystalline structures, and thus high nucleation activity, also improves the strength characteristics [109,110].

#### 3.2.4. Tests of the Strength Properties of Composite Materials

Tests of the strength properties of polypropylene composites with cellulose fillers were carried out in order to determine the influence of controlled enzymatic modification of cellulose on the strength properties of these materials. Table 7 presents the results of mechanical tests, such as: tensile strength, Young’s modulus, elongation at break and impact strength for the polymer matrix and all composite systems.

Analyzing the results obtained for the tested systems, it was found that the composite systems after the enzymatic treatment had higher values of stress at break than in the case of the polymer matrix (30.2 MPa), as well as composite materials with microcrystalline cellulose (31 MPa). Attention should be paid to obtaining higher tensile strength for systems with cellulose treated with cellulose enzymes from the microscopic fungus of the genus *Trichoderma* (approx. 36 MPa) compared to composites with filler treated with cellulase from *Aspergillus* sp. (32–34 MPa). An improvement in strength as a result of adding nanocellulose was also observed in the case of other polymer matrices, e.g., gelatin–starch [111], polyester [112], poly(butylene adipate-co-terephthalate) [113] or chitosan [114]. Moreover, improved mechanical parameters were obtained for composites containing maleated polypropylene reinforced with crystalline nanocellulose [77] and a polypropylene matrix with the addition of cellulose nanofibers in the form of a suspension with 10 wt. percentage [74].

The lowest values of Young’s modulus of elasticity were characteristic for nanocomposites whose filler was modified with cellulase from *Trichoderma reesei*, at the level of approx. 1.4 GPa and slightly higher than the unfilled polymer matrix (1.3 GPa). Higher values of the modulus of elasticity were recorded for composite materials with native cellulose and with a filler after enzymatic hydrolysis with cellulase from *Aspergillus* sp. (1.52–1.77 GPa), which may be due to a higher content of the α-PP variety. Additionally, Lee et al. [115] observed that the composite of polyamide 6 with the addition of nanocellulose was characterized by a significant increase in the modulus of elasticity compared to a polyamide matrix.

The work of Liu et al. [61] showed that the tensile strength after the introduction of cellulose nanoparticles increased by almost 15% compared to a pure polypropylene matrix (approx. 28 to 33 MPa) and a reduction in impact strength (approx. 24 to 18 J/m) was found. Liungberg et al. [73] noted that chemically obtained nanocellulose, which was additionally modified by the addition of surfactants, was responsible for obtaining an increase in tensile strength (approx. 19 to 27 MPa). Moreover, in another work [77], it was noted that CNC/polypropylene composites with the addition of a compatibilizer, were characterized by greater tensile strength by 16% (approx. 37 to 41 MPa) and a greater Young’s modulus by 30% (approx. 1180 to 1580 MPa) compared to the unfilled matrix. Comparing the results of mechanical tests achieved in other studies, it can be seen that the obtained polymer composites in our tests are characterized by significantly higher values of stress at break as well as modulus of elasticity. It is also worth noting that the composites in this study did not contain any compatibilizing agents, as in other studies, which confirms the high application potential. The explanation for this is that, as a result of enzymatic processes, a cellulose filler of nanometric dimensions was obtained, without micrometric particles. In addition, fillers with such dispersion characteristics of particles are responsible for the possibility of selectively shaping individual polymorphs of the polypropylene matrix, which in turn determines the final strength properties of the obtained composite systems.

An interesting aspect is that nanocomposites with cellulose nanofiller obtained as a result of an enzymatic reaction with the use of the enzyme of *Trichoderma reesei* are characterized by much higher elongation at break values (approx. 70%) than other analyzed composites (approx. 18–34%). Additionally, studies undertaken by El-Hadi [116] showed that the addition of nanometric cellulose to poly(lactic acid) resulted in a significant improvement in elongation, even up to 205%. However, Srivastava et al. [117] found that the addition of 3 wt. percentage nano-sized cellulose to polyvinyl alcohol resulted in an improvement in tensile strength, while the elongation at break was reduced. Moreover, obtaining these results may be caused by the presence of a much larger amount of the β-PP variant, the presence of which causes lower elasticity and greater deformability [118] than the polypropylene matrix, in which there is only the α-PP variant or the content of the β-PP is negligible.

Obtaining greater flexibility by composites with nanocellulose obtained by treatment with enzymes from *Trichoderma reesei* was also confirmed by impact tests. For this type of composite material, significantly higher impact toughness values were obtained, which amounted to approx. 38 kJ/m^2^. The remaining composite materials containing micrometric as well as nanometric cellulose obtained as a result of the enzymatic reaction with *Aspergillus* sp. were characterized by a much lower impact strength at the level of approx. 17–27 kJ/m^2^. Greater resistance to cracking under load for composite materials with filler of Cel_A-Tr and Cel_B-Tr can be explained by a higher content of nanometric particles as well as a higher content of the β-PP variety.

The conducted mechanical tests have shown that obtaining nanometric cellulose as a result of enzymatic reactions may affect the strength properties of the obtained polymer composites. However, it should be noted that only composites with the filler obtained with the use of the *Trichoderma reesei* enzyme are characterized by greater strength at break, impact strength and flexibility.

## 4. Conclusions

The use of a complex of cellulases obtained from a fungus of the genus *Trichoderma* turned out to be an effective method of obtaining cellulose of nanometric particles with a very low polydispersity. It should also be emphasized that for the first time, nanocellulose obtained as a result of controlled enzymatic hydrolysis of micrometric cellulose was used to produce polypropylene composites with a defined supermolecular structure, nucleation activity and strength characteristics.

The conducted XRD, DLS, FTIR and HPLC tests showed that the efficiency of cellulose bioconversion depends on the cellulolytic enzyme used. The highest efficiency of the enzymatic hydrolysis process was observed when using the enzyme complex from the microscopic fungus *Trichoderma reesei*, which was confirmed by obtaining more glucose, higher values of the degree of crystallinity and a greater amount of cellulose nanoparticles compared to the use of cellulolytic enzyme from *Aspergillus* sp. It is worth mentioning that by controlled enzymatic hydrolysis, it is possible to obtain cellulose with an amount of nanometric particles ranging from about 84% to 99% (depending on the type of micrometric cellulose used). Moreover, nanocelluloses obtained with the use of the *Trichoderma reesei* enzyme are characterized by lower polydispersity compared to materials obtained with the use of the enzyme from *Aspergillus* sp. It should be emphasized that a greater amount of nanometric fraction was obtained compared to the results described in other studies, which was a direct cause of obtaining higher strength of polypropylene composites than those described to date. However, it should be noted that obtaining a large amount of nanometric particles was responsible for obtaining a low recovery of nanocellulose, and a large amount of simple sugars. The use of enzymes with lower activity in the hydrolysis process contributed to a significant increase in the recovery of nanocellulose, although the obtained fraction of nanofillers was characterized by greater polydispersity and the presence of micrometric particles.

Physicochemical studies of composite materials have shown interesting relationships between the dispersion–morphological parameters of nanocelluloses and the supermolecular structure and mechanical properties of the obtained composites. Structural tests of polypropylene composites have shown that the type of cellulose nanofillers used has a significant impact on the generation of two polymorphs occurring in the polypropylene matrix—α and β. It turned out that the filler obtained as a result of the reaction using cellulase from *Trichoderma reesei* influenced the formation of a very large amount of the β-PP variant (about 40%) in composites. In addition, composites containing nanometric fraction fillers were characterized by a higher content of this polymorph in comparison to the systems containing micrometric cellulose.

Moreover, obtaining nanometric cellulose as a result of enzymatic hydrolysis with the use of the *Trichoderma reesei* enzyme had a significant effect on increasing the nucleation activity of the filler surface. Composites with nanocellulose were characterized by higher crystallization temperature values, higher phase conversion values and shorter induction times and crystallization half-times compared to composites containing micrometric cellulose and cellulose treated with cellulolytic enzyme from *Aspergillus* sp. It is also worth noting that nanocellulose showed high activity in the formation of transcrystalline structures at the polymer–filler interface.

The performed mechanical tests clearly prove the influence of the conducted enzymatic treatment of cellulose on the strength parameters of polymer composites. Composites containing cellulose treated with cellulase from *Trichoderma* were characterized by significantly higher tensile strength, lower Young’s modulus of elasticity and higher elongation at break and toughness. The obtained results perfectly correlate with structural studies, which show that composites with the highest content of the β-PP variety are characterized by greater flexibility, which is extremely important in the context of increasing the application potential of the obtained composite materials, e.g., in thermoforming packaging techniques.

To sum up, it should be emphasized that this article describes for the first time that by selecting appropriate cellulolytic enzymes in the cellulose hydrolysis process, it is possible to control the structure and dispersion and morphological parameters of the obtained cellulose nanofillers. In addition, the obtained cellulose nanofibers with defined parameters can be used for the production of polymer composites with a strictly defined polymorphic structure, which in turn may influence future decision making about obtaining materials with controllable properties, e.g., high flexibility, enabling the thermoforming process of packaging.

## Figures and Tables

**Table 1:** Designations of obtained cellulose materials.

Sample	Material
Cel_A	Sigmacell Type 20
Cel_A-Tr	Sigmacell Type 20—*Trichoderma reesei*
Cel_A-Asp	Sigmacell Type 20—*Aspergillus* sp.
Cel_B	Sigmacell Type 101
Cel_B-Tr	Sigmacell Type 101—*Trichoderma reesei*
Cel_B-Asp	Sigmacell Type 101—*Aspergillus* sp.

**Table 2:** Glucose concentration (in mg/mL) in post-reaction solutions (n.d.—not determined).

Sample	Glucose Concentration (mg/mL)	Recovery of Nanocellulose (%)
Cel_A	n.d.	n.d.
Cel_A-Tr	27.31	18
Cel_A-Asp	3.66	89
Cel_B	n.d	n.d
Cel_B-Tr	17.10	49
Cel_B-Asp	4.30	87

**Table 3:** The results of the X-ray diffraction pattern of native celluloses and celluloses hydrolyzed with enzymes.

Sample	Degree of Crystallinity (%)
Cel_A	49
Cel_A-Tr	56
Cel_A-Asp	43
Cel_B	29
Cel_B-Tr	51
Cel_B-Asp	32

**Table 4:** Dispersive properties of modified celluloses.

Sample	Z-Average (nm)	PDI
Cel_A-Tr	75	0.105
Cel_A-Asp	505	0.277
Cel_B-Tr	76	0.181
Cel_B-Asp	135	0.217

**Table 5:** The content of the β-PP form in composite systems and polypropylene matrix.

Sample	Content of the β-PP Form (%)
PP	10
PP + Cel_A	20
PP + Cel_A-Tr	41
PP + Cel_A-Asp	22
PP + Cel_B	17
PP + Cel_B-Tr	40
PP + Cel_B-Asp	29

**Table 6:** The results of the differential scanning calorimetry and the polarizing interference microscopy for materials with PP matrix and cellulose fillers (n.d.—not determined).

Sample	Half-Time of Crystallization (min)	Temperature of Crystallization (°C)	Degree of Crystallinity (%)	Induction Time (min)	Transcrystalline Layer Growth Rate (μm/min)
PP	2.7	113.3	39	n.d.	n.d.
PP + Cel_A	2.2	122.5	20	5	2.2
PP + Cel_A-Tr	1.4	128.2	20	3	2.8
PP + Cel_A-Asp	2.1	120.3	21	11	1.2
PP + Cel_B	2.0	122.1	22	13	1.5
PP + Cel_B-Tr	1.4	129.2	25	3	3.2
PP + Cel_B-Asp	1.8	127.6	20	3	2.4

**Table 7:** Mechanical tests for composite materials and polypropylene matrix.

Sample	Tensile Strength (MPa)	Modulus (GPa)	Elongation at Break (%)	Impact Strength (kJ/m^2^)
PP	30.2 (0.18)	1.31 (0.08)	342 (21.1)	51.8 (0.89)
PP + Cel_A	31.5 (0.37)	1.75 (0.20)	18.2 (3.8)	18.4 (2.31)
PP + Cel_A-Tr	35.8 (0.20)	1.43 (0.13)	72.5 (5.6)	37.6 (1.24)
PP + Cel_A-Asp	32.4 (0.33)	1.70 (0.21)	21.6 (3.1)	20.4 (2.04)
PP + Cel_B	31.1 (0.41)	1.77 (0.19)	15.7 (3.9)	16.9 (2.75)
PP + Cel_B-Tr	36.1 (0.26)	1.46 (0.11)	69.8 (4.3)	38.1 (1.56)
PP + Cel_B-Asp	34.4 (0.23)	1.52 (0.14)	34.7 (3.6)	27.3 (1.94)

## Data Availability

The data reported in this study can be available by request from the authors.
